# Tumor Evolution in Space: The Effects of Competition Colonization Tradeoffs on Tumor Invasion Dynamics

**DOI:** 10.3389/fonc.2013.00045

**Published:** 2013-03-06

**Authors:** Paul A. Orlando, Robert A. Gatenby, Joel S. Brown

**Affiliations:** ^1^Biometry Research Group, Division of Cancer Prevention, National Cancer InstituteRockville, MD, USA; ^2^Department of Radiology, Moffitt Cancer CenterTampa, FL, USA; ^3^Department of Biological Sciences, University of Illinois at ChicagoChicago, IL, USA

**Keywords:** tumor invasion, spatial ecology, competition colonization tradeoff, partial differential equation model, spatial selection

## Abstract

We apply competition colonization tradeoff models to tumor growth and invasion dynamics to explore the hypothesis that varying selection forces will result in predictable phenotypic differences in cells at the tumor invasive front compared to those in the core. Spatially, ecologically, and evolutionarily explicit partial differential equation models of tumor growth confirm that spatial invasion produces selection pressure for motile phenotypes. The effects of the invasive phenotype on normal adjacent tissue determine the patterns of growth and phenotype distribution. If tumor cells do not destroy their environment, colonizer and competitive phenotypes coexist with the former localized at the invasion front and the latter, to the tumor interior. If tumors cells do destroy their environment, then cell motility is strongly selected resulting in accelerated invasion speed with time. Our results suggest that the widely observed genetic heterogeneity within cancers may not be the stochastic effect of random mutations. Rather, it may be the consequence of predictable variations in environmental selection forces and corresponding phenotypic adaptations.

## Introduction

Competition-colonization tradeoffs underlie an important mechanism of coexistence in ecological communities with spatial variation of competitor abundances (Tilman, 1994). In these communities, some species excel at colonizing unoccupied space whereas others excel at competing within already occupied space. But, no species simultaneously excels at both. Ecologists have demonstrated competition colonization tradeoffs in a number of communities (e.g., birds: Rodríguez et al., 2007, ants: Stanton et al., 2002, plants: Turnbull et al., 2004). They can be important in structuring ecological communities (e.g., Turnbull et al., 1999; Cadotte et al., 2006).

Competition colonization tradeoffs may also play an important role in the ecological and evolutionary dynamics of population invasions and range expansions. Researchers have noted that selective pressures at an invasion front could be markedly different than selective pressures at the core of an invasion (e.g., Phillips, 2009; Burton et al., 2010). Evolutionary ecologists have shown that phenotypic change by natural selection occurs during species invasions and is critical for understanding invasion dynamics (e.g., Simmons and Thomas, 2004; Broennimann et al., 2007; Barrett et al., 2008).

A well-known example of eco-evolutionary dynamics is the invasion and spread of the cane toad (*Bufo marinus*) across northern Australia. Detailed examination of the spreading population demonstrates two divergent phenotypes based on selection for colonization along the invasion front (Phillips et al., 2006). The colonizing phenotype has longer legs, moves more often, and is found near the front of the invasion. Phillips (2009) has shown that the phenotype at the invasion front tend to be *r*-selected, in that they reproduce sooner than toads in the core. Evidence suggests that a tradeoff for increased dispersal may be manifest in increased spinal stress and arthritis (Brown et al., 2007).

We highlight the notion that tumor invasion parallels the process of population invasion into novel habitats and subsequent range expansion. Accordingly, concepts and modeling from ecology and evolution can be applied to understand the ecological and evolutionary dynamics of tumors. While the modern paradigm of cancer biology sees cancer as arising because of cell level selection pressures, oncologists have largely neglected the role of ecology in determining these selection pressures and subsequent evolution (Gatenby, 2012). Integrating these viewpoints has the potential to further our understanding of the growth and invasion of tumors.

There is clear evidence of evolutionary processes within clinical cancers resulting in multiple genetically distinct clones (Yachida et al., 2010; Gerlinger et al., 2012). However, this is typically attributed to random mutations that result in an overall proliferative advantage rather than local adaptations to specific environmental selection forces. Selection in tumors could be markedly different at the tumor host interface than within the host. Moreover, there is evidence that suggests the presence of both colonization and competition phenotypes among cancer cells within a tumor. For example, invadopodia are actin rich invasive cell membrane protrusions that degrade the extracellular matrix (Weaver, 2006). Invadopodia have been observed in a wide range of cancers and appear to confer invasion potential. In contrast, the phenotypes of many cancer cells appear to promote the development of a local tumor infrastructure. For example, vascular endothelial growth factor (VEGF) is a protein secreted by many tumor cells and promotes tumor vascularity and blood flow by inducing growth and movement of endothelial cells (Carmeliet and Jain, 2000; Goodsell, 2003). The former phenotype may arise due to selection pressures at the invasion front, and the latter may arise due to selection pressures within the interior of the tumor.

In this article we use partial differential equation (PDE) models that are spatially, ecologically, and evolutionarily explicit to explore the effects of competition colonization tradeoffs on the evolution of tumors. PDE models of population growth in space have a history in both the fields of ecology (e.g., Holmes et al., 1994) and tumor biology (e.g., Chaplain et al., 2006; Eikenberry et al., 2009). Our approach is novel in that we explicitly model a phenotypic distribution of the cancer cells (also, see Benichou et al., 2012; Bouin et al., 2012). In our model, cancer cells are distributed in physical space and phenotype space. As such our model may better reflect the ecological and evolutionary dynamics of tumor invasion by incorporating population dynamics and heritable phenotypic changes.

We use our models to investigate four important questions relevant to the eco-evolutionary dynamics of range expansions and tumor biology.

When does cell motility evolve? Models of range expansion show that motility can evolve, and this has been demonstrated in general population models and tumor specific models (e.g., Gerlee and Anderson, 2009; Aktipis et al., 2012). We use our models to reaffirm these results and to explore when and if cell motility evolves.

Does the type of movement matter? Previous work with spatial PDE models has demonstrated that spatial heterogeneity with temporal homogeneity selects against diffusive movement, but can select for directed adaptive movement (Dockery et al., 1998; Cantrell et al., 2006). However, there has not been an analysis comparing different movement types in models of range expansion. We use our models to compare the effects of different types of movement rules on the overall eco-evolutionary dynamics.

Does the evolution of cell motility result in phenotypic differentiation in space? The cane toads are clearly an example of phenotypic divergence in space. However, recent theoretical work by Shine et al. (2011) has shown that selection is not necessary for phenotypic divergence in a spatial context. Rather spatial assortment of phenotypes can simply be a consequence of the fact that faster moving phenotypes tend to move to the invasion front, and the slower moving phenotypes tend to stay in the core, and this facilitates assortive mating. Theoreticians have demonstrated the effect of spatial sorting in PDE models of range expansion (Benichou et al., 2012; Bouin et al., 2012). We use our models to ask if selection for motility results in phenotypic divergence in space. More specifically, we investigate whether a competition colonization tradeoff is required for this type of landscape scale coexistence.

Does invasion speed accelerate? The speed of the cane toad invasion has accelerated, by as much as five times in a half century (Phillips et al., 2006, 2007). Researchers attribute this acceleration to the evolution of a more specialized colonizer phenotype. Individual toads have been shown to move longer distances per unit time in recent times as compared to historic records. Theory also predicts accelerated invasion speed with the evolution of dispersal (Travis and Dytham, 2002). Thus, we explore with our models whether the evolution of motility results in accelerated invasion speed.

## Model Description

We develop two spatially and evolutionarily explicit PDE models to explore tumor invasion with a competition colonization tradeoff. The models contrast two extreme perspectives on tumor dynamics. In the first model, cancer cells invade the surrounding microenvironment and subsequently reach a carrying capacity. In the second model, cancer cells invade the surrounding microenvironment and subsequently destroy the environment, resulting in local extinction of the cancer cells. In both models, cancer cells are characterized by their phenotype and location in physical space. Thus, we include a phenotypic dimension (*w*), which describes a phenotypic distribution (Cohen, 2009) of cancer cells. To model a competition colonization tradeoff, we assume that increased *w* corresponds to increases in a cell’s ability to move in physical space and decreases its ability to compete for resources. Numerical solutions to the models describe the time evolution of the phenotypic distribution of cells in space. Mutation and differential success of phenotypes results in ecological and evolutionary dynamics. Cell movement produces spatial ecological dynamics.

### Model 1 – a habitat–continuum tumor model

With the first model, we consider an ecological situation where cancer cells invade the surrounding environment and engineer the environment, such that it is a suitable habitat. This model is a phenomenological representation of angiogenesis and other types of environmental engineering by the cancer cells. We model a logistically growing population of cancer cells (*c*) in one-dimensional space (*x*), with phenotype (*w*). We consider two different versions of the model; one for the evolution of random movement and one for the evolution of directed movement. The corresponding PDEs are given by

(1) $$\begin{array}{llll} \frac{\partial c\left(x,w,t\right)}{\partial t} & =\lambda c\left(\frac{K\left(w\right)-T_{c}}{K\left(w\right)}\right)+\mu \left(w\right)\frac{\left(\partial^{2}c\right)}{\partial x^{2}} & & \\ & \;-\frac{\partial }{\partial x}\left(\chi c\frac{\partial F}{\partial x}\right)+M\left(w\right). & \end{array}$$

(2) $$\begin{array}{llll} \frac{\partial c\left(x,w,t\right)}{\partial t} & =\lambda c\left(\frac{K\left(w\right)-T_{c}}{K\left(w\right)}\right)+\mu \frac{\partial^{2}c}{\partial x^{2}} & & \\ & \;-\frac{\partial }{\partial x}\left(\chi \left(w\right)c\frac{\partial F}{\partial x}\right)+M\left(w\right). & \end{array}$$

The first term in the equations describes standard logistic population growth, with an intrinsic growth rate λ, and carrying capacity *K*(*w*). *T_c_* represents the total cell density at a spatial position *x*. *T_c_* is calculated by integrating over the phenotypic dimension, giving $T_{c}=\int_{0}^{1}c\left(x,w\right)dw.$

The second and third terms are derived by Fick’s first and second laws of flux. The second term describes random movement in space, as characterized by a Laplacian operator scaled by the cell motility coefficient μ. The third term describes directed cell movement in space via the spatial fitness gradient (∂*F*/∂*x*) and proportional to the tactic sensitivity coefficient χ. The tactic sensitivity coefficient scales the tendency of cells to move in response to a chemical gradient. Here, the fitness function is defined as the per capita growth rate of cells in the absence of cell movement or mutation:

$$F=\lambda \left(\frac{K\left(w\right)-T_{c}}{K\left(w\right)}\right).$$

Since the fitness term is density dependent, the cells “adaptively” move to areas with lower cell densities. The fourth and final term in the model describes mutation or movement in phenotype space (see below for description).

The competition colonization tradeoff enters the model through the carrying capacity *K*(*w*) and the cell movement parameters. To explore the effect of the evolution of different cell movement rules on invasion dynamics, we model the evolution of cell movement in two different ways (Eqs 1 and 2). The phenotypic variable *w* either increases the cell motility coefficient μ, μ = ρ_1w_ Eq. (1), or increases the tactic sensitivity coefficient χ, χ = ρ_1w_ Eq. (2). Throughout, we refer to the former as random cell movement and the latter as directed cell movement. In both cases, increasing *w* necessarily decreases cell competitiveness by decreasing the carrying capacity of a specific phenotype,

$$K\left(w\right)=\kappa \;\exp\;\left(-\rho_{2}w\right).$$

Following Cohen (2009), we use a discrete function (Eq. 3) to describe mutation with regard to a continuous phenotypic trait (*w*). The *B* function describes the per capita birth rate of a particular phenotype, with ε describing the mutational step size. As in Cohen (2009) we assume for simplicity that each phenotype has a constant per capita birth rate λ, such that the negative part of the per capita logistic growth equation represents death rates. This simplification then leads to Eq. 4. We use second order Taylor series approximations of the terms in Eq. 3 to convert the discrete equation into a continuous approximation. Equation 4 shows the second order Taylor series approximation to Eq. 3. We use Eq. 5 as the mutation term in the model.

(3) $$\begin{array}{llll} M^{\prime }\left(w\right) & =\frac{1}{2}\eta [B\left(w+\varepsilon \right)c\left(w+\varepsilon \right) & & \\ & \;+B\left(w-\varepsilon \right)c\left(w-\varepsilon \right)-2B\left(w\right)c\left(w\right)] & \end{array}$$

(4) $$\begin{array}{lll} M^{\prime }\left(w\right) & =\frac{1}{2}\eta \lambda \left[c\left(w+\varepsilon \right)+c\left(w-\varepsilon \right)-2c\left(w\right)\right] & \end{array}$$

(5) $$\begin{array}{lll} M\left(w\right) & =\frac{1}{2}\eta \lambda \varepsilon^{2}\frac{\partial^{2}c}{\partial w^{2}} & \end{array}$$

### Model 2 – a habitat-destruction tumor model

Our second model considers an ecological scenario where cancer cells invade and subsequently destroy the microenvironment. This model represents tumors with a significant necrotic core. We use a modified version of the haptotaxis model introduced by Anderson (2005). The system of PDEs is given by

(6) $$\begin{array}{llll} \frac{\partial c\left(x,y,w,t\right)}{\partial t} & =\nu Z\left(p\right)c-D\left(w\right)c+\mu \Delta_{x,y}c & & \\ & \;-\nabla_{x,y}\left(\chi \left(w\right)c\nabla_{x,y}m\right)+M\left(w\right) \end{array}$$

(7) $$\begin{array}{llll} \frac{\partial c\left(x,y,w,t\right)}{\partial t} & =\nu Z\left(p\right)c-D\left(w\right)c+\mu \Delta_{x,y}c & & \\ & \;-\nabla_{x,y}\left(\chi \left(w,m\right)c\nabla_{x,y}p\right)+M\left(w\right) & \end{array}$$

(8) $$\begin{array}{lll} \frac{\partial m\left(x,y,t\right)}{\partial t} & =-\alpha mT_{c} & \end{array}$$

(9) $$\begin{array}{ll} \frac{\partial p\left(x,y,t\right)}{\partial t} & =\gamma m-\sigma p-Z\left(p\right)T_{c}+\omega \Delta_{x,y}p \end{array}$$

This model includes, cancer cell density (*c*), extracellular matrix density (*m*), and oxygen concentration (*p*) as state variables. The model assumes that cancer cells use extracellular matrix macromolecules for movement, and in the process, degrade these molecules. Furthermore, the matrix molecules produce oxygen, which the cancer cells depend on for reproduction. Thus, as cancers cells invade the surrounding environment they leave a wake of habitat-destruction by degrading the extracellular matrix and their oxygen supply. We assume that oxygen uptake by the cancer cells is described by a saturating function *Z*(*p*) = ψ*p*/(þeta + *p*), where ψ is the maximum uptake rate and θ is the half saturation concentration of oxygen. ν Is the conversion efficiency of consumed oxygen to new cancer cells. δ Is the per capita death rate of cancer cells. As in the previous model, μ and χ represent the cell motility coefficient and the tactic sensitivity coefficient respectively. *M* represents mutation, which we modify slightly from before (see below). Equation 6 shows that the matrix macromolecules decline from an initial abundance. In Anderson’s original model, the degradation of the matrix was mediated through a matrix degradation protein that the cancer cells produced. For simplicity, here we consider that the cancer cells directly degrade the matrix molecules. Empirically, this mechanism may be captured by invadopodia for instance. α Describes the per capita rate at which cancer cells contact and degrade matrix molecules. Finally, the rate of change of oxygen concentration is linearly dependent on matrix molecules, where γ is the per molecule production of oxygen (Eq. 9), and σ is the per capita degradation rate of oxygen. Oxygen also declines through consumption. ω Is the diffusion coefficient for oxygen.

To explore the effects of the evolution of different cell movement rules on invasion dynamics, we model two different versions of the tradeoff. Since the cells use the matrix molecules for movement, we assume that the cell motility coefficient is small and that the main mechanism of cell movement is through haptotaxis or chemotaxis. In both versions, the cost of increased tactic sensitivity is mediated through increased per capita death rate of cancer cells. Thus, *D* = δ + ρ_2w_. Where δ is the minimum per capita death rate, and ρ_2_ scales the effect of increased cell motility on cell death rate. In the haptotaxis version of the model Eq. (6), we assume that directed cell movement is in the direction of increasing matrix molecules, with a speed proportional to the haptotactic coefficient, which is a function of the cells phenotype. χ = ρ_1w_. In the chemotactic version of the model Eq. (7), directed cell movement is in the direction of increasing oxygen concentration. The chemotactic sensitivity coefficient is a function of the density of matrix macromolecules and cell phenotype. χ = *m*ρ_1w_. This models a situation where cells move toward areas with higher oxygen concentrations, but depend on matrix macromolecules for movement. As with the first model, this second version considers adaptive movement of cells.

The mutation term in this model is slightly different than the last model, since we have a specific function that describes the birth rate of each phenotype. The birth rate, *B*(*w*) is given by the first terms of Eqs 6 and 7, *B*(*w*) = ν*Z*(*w*)*c*(*w*), substituting this into Eq. 3 above, and performing the Taylor series approximation as described above, gives *M*(*w*) = (1/2)ηε^2^∂^2^*B*/∂*w*^2^ for the mutation term in the model.

In the first model, we consider a spatial line of 10 mm. In the second model, we consider a spatial area of 10 mm × 10 mm. We used Neumann (no flux) boundary conditions for both the spatial and the phenotypic boundaries.

## Numerical Analysis

We analyzed both models through numerical simulations, for which we used the method of lines approach (Schiesser and Griffiths, 2009). We used upwind spatial finite differences for the tactic terms. Anderson’s (2005) original model is particularly difficult to solve numerically. We confirmed the validity of our scheme, by solving Anderson’s original model and comparing our results to those of Walker and Webb (2007) and Chertock and Kurganov (2008). We found our results to be in good agreement with those of others.

For the first model, we used two different initial conditions. For an initial condition of mostly the competitor phenotype, we used

$$c\left(x,w,0\right)=5\max\left\{0,\left(0.3-{\left(x-5\right)}^{2}\right)\right\}*\exp\left(-100*w\right).$$

For an initial condition with mostly colonizers, we used:

$$c\left(x,w,0\right)=5\max\left\{0,\left(0.3-{\left(x-5\right)}^{2}\right)\right\}*\exp\left(-100*\left(1-w\right)\right).$$

Both initial conditions represent a small population of cancer cells in the center of the spatial domain.

We used the following parameters for first model:

λ = 0.5, κ = 1e–5, η = 1e−3, ρ_2_ = 1. ρ_1_ = 1e−2 and μ = 1e−5 for the evolution of chemotactic sensitivity. χ = 1e−4 and ρ_1_ = 1e−3 for the evolution of cell motility.

For the second model, we used the following initial conditions for both versions of the model:

$$\begin{array}{llll} c\left(x,y,w,0\right) & =500\max\left\{0,\left(0.3-{\left(x-5\right)}^{2}+{\left(y-5\right)}^{2}\right)\right\} & & \\ & \;\times \exp\left(-100*w\right). & & \\ m\left(x,y,0\right) & =0.05\cos\left(\left(\pi x^{2}\right)∕20\right)*\sin\left(\left(\pi y^{2}\right)∕20\right)+0.1. & & \end{array}$$

*p*(*x*, *y*, 0) = 5*m*(*x*, *y*, 0). These initial conditions are similar to those of Walker and Webb (2007). They represent a small population of cancer cells in the center of the domain and a heterogeneous spatial distribution of ECM and oxygen.

We used the following parameters for the second model:

μ = 1e−5, α = 1e−2, σ = 0.1, γ = 30, ω = 5e−2, η = 1e−3, δ = 0.2, ρ_1_ = 0.1, ρ_2_ = 5e−2, þeta = 0.5, ν = 10, ψ = 0.1.

To investigate selection for cell motility we compare three evolutionary situations with both models: (1) there is no cost to increased cell motility (no tradeoff, ρ_2_ = 0), (2) there is a cost, but no benefit – variation in the phenotypic variable (*w*) does not correspond to increased cell motility (i.e., ρ_1_ = 0), and (3) there is a cost to increased cell motility (tradeoff, ρ_1_ > 0, ρ_2_ > 0). The strongest selection for cell motility should occur when there is no cost. On the contrary, in the situation, where the phenotypic variable (*w*) does not correspond to increased cell motility, there is a cost, but no benefit. This situation is considered because mutation and selection create a phenotypic distribution. Thus, even if cell motility is selected against (i.e., *w* = 0 is optimum) there will still be an increase in the mean value of cell motility due to mutation. So this serves as a null case for comparison. When there is both a cost and a potential benefit, then the trait should increase in the population beyond when there is just a cost, but below the value when there is no cost.

## Results

### Model 1 – competition colonization tradeoffs in a habitat–continuum tumor model

**Figure 1:**
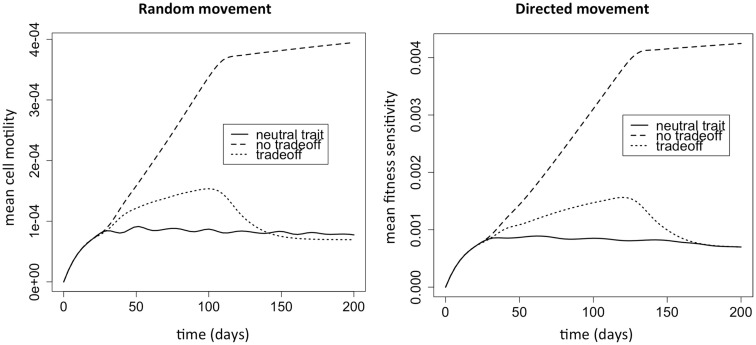
**Selection for motility when the phenotypic variable affects random versus directed movement**. The “neutral trait” has only a cost, but does not actually correspond to variation in motility. Therefore, it simply shows the mutation selection balance when the trait has no benefit and thus there is no selection for the trait (ρ_1_ = 0). At the other extreme, the “no tradeoff” scenario corresponds to a situation where there is no cost to increased cell motility (ρ_2_ = 0). Finally, “tradeoff” is a situation where increased motility comes at a cost of decreased competitiveness (ρ_1_ > 0 and ρ_2_ > 0). Note that the *y* variable in the plots is *k*_1w_, which represents the cell motility coefficient or the tactic coefficient. When there is no benefit to increasing the phenotypic variable *w* (ρ_1_ = 0), we set ρ_1_ = 0.1 to plot the variable for comparison (solid lines); although the variable does not actually correspond to variation in cell motility it does reflect changes in *w*.

We first investigate natural selection for cell motility. We do this by comparing the three evolutionary situations discussed above. The strongest selection pressure for cell motility should occur when there is no cost to increased cell motility. When there is only a cost and no benefit to the trait, then there should be selection against the trait. In this case, the fittest phenotype is the most competitive, and the distribution will simply reflect a mutational spread around this most fit phenotype. When there is a tradeoff, and motility is selected for, the mean trait value should intuitively lie somewhere between these extremes. Figure 1 shows the dynamics of the mean evolutionary trait for the three scenarios. After around 30 days, sufficient phenotypic variation has accumulated and the population size has achieved a size that manifests a positive selection for motility. After close to 100 days, most of the space has been colonized, and there is selection against motility and in favor of competition instead.

**Figure 2:**
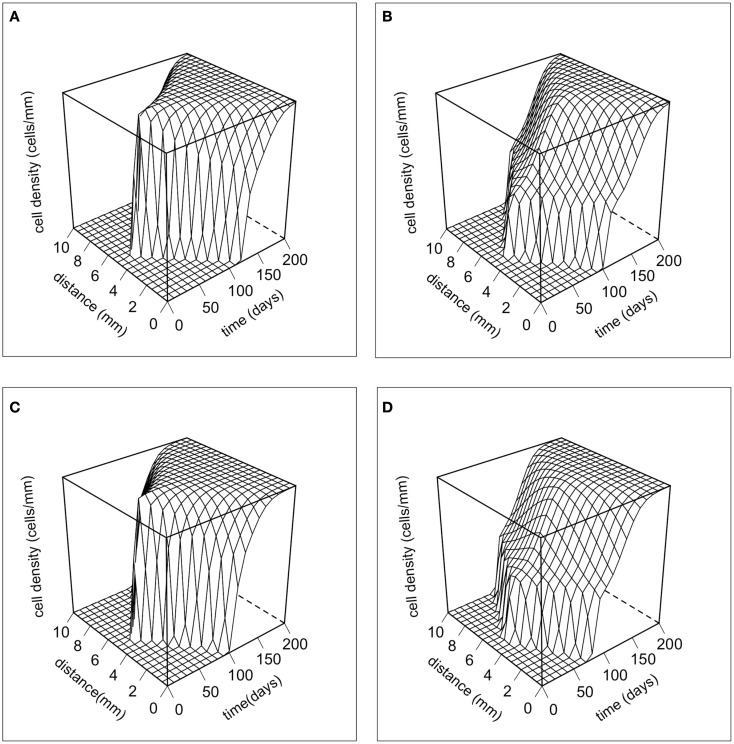
**The dynamics of the total cancer cell density in space and time**. The total cancer cell density (*T*_c_) at a particular spatial location is integrated over the phenotypic dimension. **(A)** Random cell movement with an initially competitive phenotype distribution. **(B)** Random cell movement with an initially motile phenotypic distribution. **(C)** Directed cell movement with an initially competitive phenotypic distribution. **(D)** Directed cell movement with an initially motile phenotypic distribution.

Figure 2 shows the dynamics of the total cancer cell density in time and space. There are no major differences in the dynamics produced by the two different movement rules. However, there are large differences between the phenotypic initial conditions. When the majority of the population is initially composed of strong competitors, the population increases rapidly, and then begins to spread laterally. When the initial composition of the population is mostly motile cells, the population first spreads rapidly in space and then grows up to carrying capacity. The dynamics of our model are characterized by traveling waves of cancer cells in physical space (e.g., Murray, 2003).

**Figure 3:**
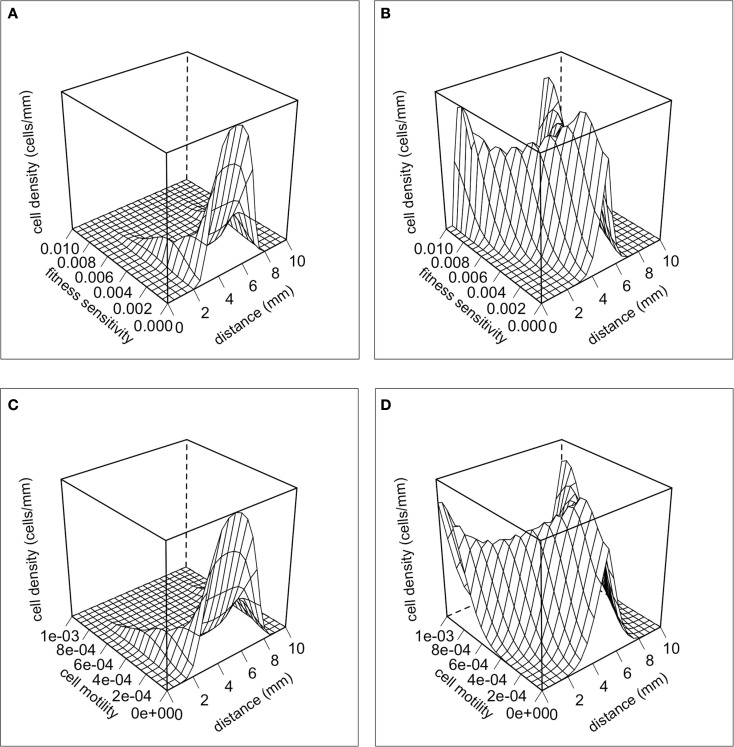
**The distributions of cells in physical space and phenotypic space at *t* = 100 days**. **(A)** Random cell movement with an initially competitive phenotype distribution. **(B)** Random cell movement with an initially motile phenotypic distribution. **(C)** Directed cell movement with an initially competitive phenotypic distribution. **(D)** Directed cell movement with an initially motile phenotypic distribution.

Selection for motility should be occurring at the margins of the tumor, and thus this can potentially create phenotypic divergence in space. Figure 3 shows snapshots of the distribution of cancer cells in physical space and in phenotype space. There is a clear pattern of phenotypic divergence in space, with the evolution of both random and directed movement and with both initial conditions. This pattern still exists without a tradeoff (*k*_1_ = 0). However, in the absence of a tradeoff the phenotypic differentiation in space is not as well defined. This is because motile phenotypes are not selected against in the core of the tumor.

**Figure 4:**
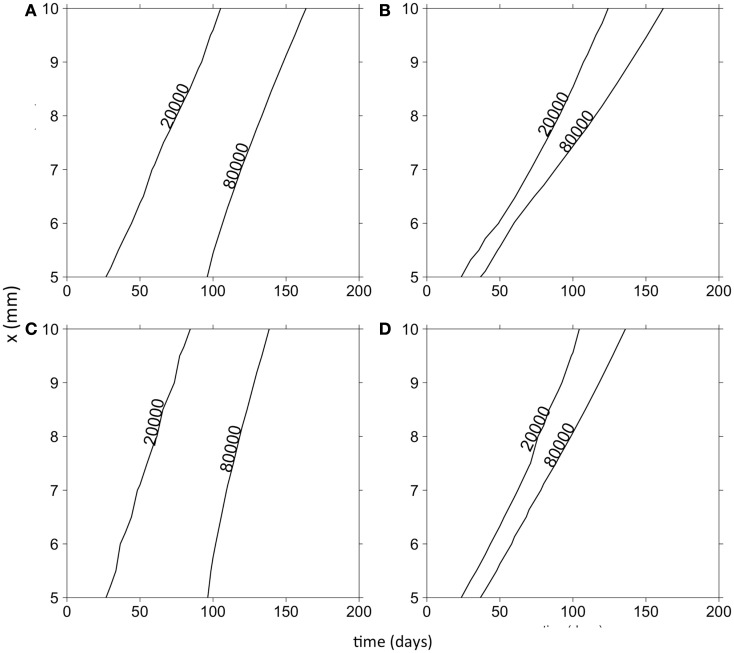
**Contour plots of the total cancer cell density in time and space**. **(A)** Random cell movement with an initially competitive phenotype distribution. **(B)** Random cell movement with an initially motile phenotypic distribution. **(C)** Directed cell movement with an initially competitive phenotypic distribution. **(D)** Directed cell movement with an initially motile phenotypic distribution.

**Figure 5:**
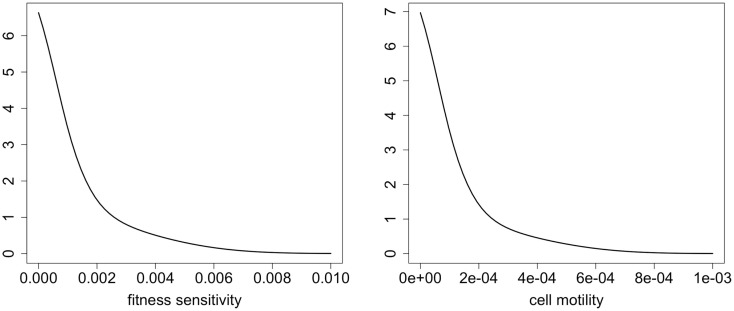
**Normalized phenotypic distributions for directed and random movement at *t* = 100 days**. These distributions are integrated over space to include the entire spatial domain.

Finally, we were interested in how the evolution of cell motility would affect invasion speed. To this end, Figure 4 shows contours of cancer cell densities in space and time. The invasion speed is calculated as the slopes of the contour lines. The figure shows that in general, the evolution of cell motility produces linear invasion speeds over time. There are only slight non-linearities. As we have shown, mean cell motility is increasing over time due to natural selection. Invasion speed should increase with the cell motility and with the chemotactic coefficients. This is shown by the fact that the invasion speed is much quicker if the cell population is initially composed of highly motile cells (Figures 4A,C). However, the phenotypic distributions tend to obscure the effect of increasing cell motility on invasion speed. This occurs because once an area is crowded with cells; there is selection to invade adjacent un-crowded areas. As cells invade the adjacent areas, the fittest phenotype is the best competitor. Because of this, a wide range of phenotypes can coexist in space. Figure 5 shows the normalized phenotypic distributions at 100 days. The distributions are wide and skewed toward the competitors. So even though mean motility increases over time, the variance obscures this signal for the population as a whole. Even when there is no tradeoff, and thus stronger selection for motility, invasion speeds remain relatively constant over time.

### Model 2 – competition colonization tradeoffs in a habitat-destruction tumor model

**Figure 6:**
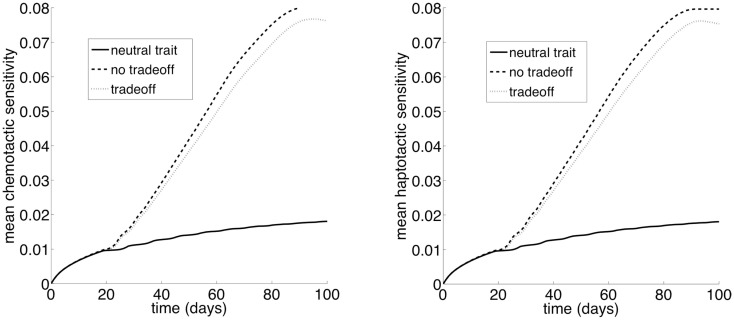
**Selection for motility when chemotactic versus haptotactic movement evolves**. The “neutral trait” has only a cost, but does not actually correspond to variation in motility. Therefore, it simply shows the mutation selection balance when the trait has no benefit and thus there is no selection for the trait (ρ_1_ = 0). At the other extreme, the “no tradeoff” scenario corresponds to a situation where there is no cost to increased cell motility (ρ_2_ = 0). Finally, “tradeoff” is a situation where increased motility comes at a cost of decreased competitiveness (ρ_1_ > 0 and ρ_2_ > 0). Note that the *y* variable in the plots is *k*_1w_, which represents the cell motility coefficient or the tactic coefficient. When there is no benefit to increasing the phenotypic variable *w* (ρ_1_ = 0), we set ρ_1_ = 0.1 to plot the variable for comparison (solid lines); although the variable does not actually correspond to variation in cell motility it does reflect changes in *w*.

The second model is fundamentally different from the first in that there is no permanent niche for competitors in this model. Instead, the environment is consumed and destroyed as the cancer cells advance and spread. Therefore, selection for motility should be strong, since it is the only niche for the cells. Figure 6 shows that there is selection for motility. The lines in the plot correspond to the same three evolutionary scenarios we considered with model 1. Given the parameters we chose, there is strong selection for motility. In this model, the cells do not reach a carrying capacity, and so there is not a strong reversal of selection once the space is filled.

**Figure 7:**
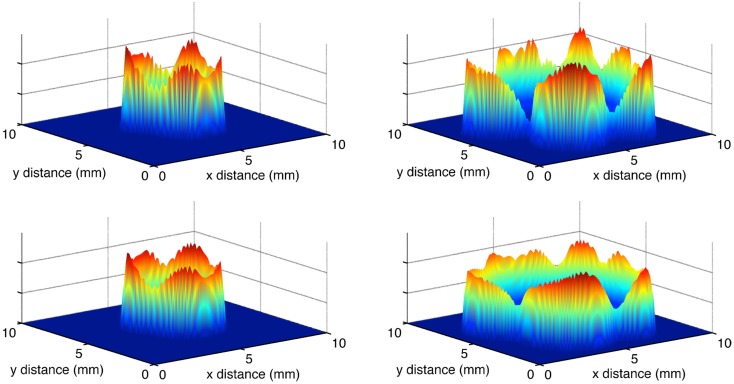
**Snapshots of the total normalized cancer cell density in two-dimensional physical space**. The left and right panels show *t* = 50 and *t* = 75 days, respectively. The top and bottom panels show the evolution of chemotaxis and haptotaxis, respectively.

As in the habitat–continuum model, the two different movement rules produce very similar tumor invasion dynamics. Figure 7 shows snapshots in time of the tumor cell densities in two dimensional physical space. As the dynamics proceed, there is an expanding wavefront of cancer cells in physical space. Eventually, the cancer cells destroy the ECM and their oxygen supply. Thus, the model reaches an equilibrium with zero cancer cells, ECM, or oxygen.

In this model there is no clear spatial coexistence of phenotypes. Due to the ephemeral nature of oxygen following invasion into a new area, phenotypes that move less frequently or slower are less fit.

**Figure 8:**
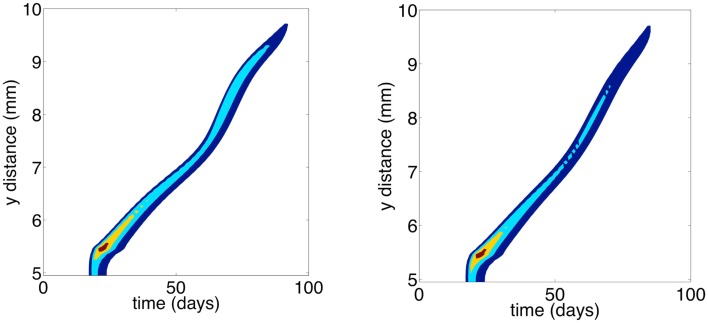
**Contour plots showing total cancer cell densities in space and time**. Blue corresponds to lower cell densities and red corresponds to higher cell densities. The *x* dimension is fixed at 5 mm. The left and right panels show the evolution of chemotaxis and haptotaxis, respectively.

**Figure 9:**
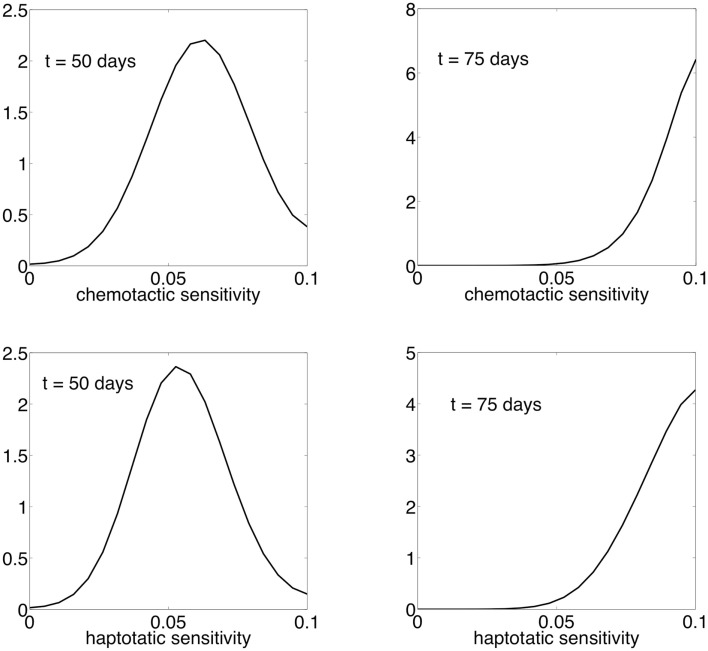
**Normalized phenotypic distributions for the evolution of chemotaxis and haptotaxis at *t* = 50 and *t* = 75 days**. The evolution of chemotaxis and haptotaxis are shown in the top and bottom panels, respectively.

Figure 8 shows the contours of total cancer cell densities in time and space. In this plot, we fix the *x* dimension to the center of the domain. The thin distribution of cancer cell densities at any time show how the cancer cells spread into an area and subsequently decline as the matrix molecules are degraded. The contours clearly show that there is an acceleration of invasion speed. This happens because there is stronger and more consistent selection for motility. Because of this, there are bigger fitness differences maintained between the phenotypes, and phenotypic variation is reduced. Figure 9 shows the phenotypic distributions for the evolution of the two different movement types at *t* = 100 days. In this case, the phenotypic variance is much reduced compared to the results of the habitat–continuum model.

## Discussion

Competition colonization tradeoffs are commonly observed in ecological communities. Furthermore, during biological invasions the populations in the leading edge adapt to different selection forces compared to those in the geographic core (e.g., Phillips et al., 2006). We address the influence of competition colonization tradeoffs on tumor invasion dynamics, since tumors dynamics in many ways parallel species invasions and range expansions into new habitats. We used two different models. The habitat–continuum tumor model sees the tumor has having a continuum from interior to edge habitats. Due to angiogenesis and other “ecological engineering,” regions of the tumor interior remain suitable habitat for the cancer cells. The habitat-destruction tumor model sees the cancer cells as “consuming” the environment. This creates a tumor with a necrotic interior and an expanding edge.

Both of our models clearly predict that evolution of cell motility. Furthermore, this evolution is mainly due to natural selection, since the motile phenotypes increased in abundance relative to other phenotypes in the population. Many other researchers have shown that motility is selected for during population invasion into new habitats. For example, Aktipis et al. (2012) recently showed that cell motility evolves in response to local environmental degradation, and may be a co-adaptation or consequence of altered cell metabolism.

We also found that in general the evolution of different types of cell movement has almost no effect on the global dynamics of the invasion. The more adaptive movement will likely be evolutionarily favored, but we predict that this will have very little impact on the overall invasion dynamics. However, if evolution affects haptotaxis or chemotaxis, and the underlying spatial distribution of the molecules, which direct movement are sufficiently different, then it is plausible that the evolution of different movement rules may produce drastically different invasion dynamics. In the tumor specific model we considered, oxygen is produced by the matrix macromolecules and as a consequence their spatial distributions are similar and thus haptotaxis and chemotaxis produce similar results.

We did find important differences between the habitat–continuum and the habitat-destruction models in terms of tumor invasion dynamics and phenotypic evolution. In the habitat–continuum tumor model, we found that the invasion speed was relatively linear over time. This occurred because there is relatively weak and ephemeral selection for cell movement at a particular location, which allows for the coexistence of many phenotypes and hence large diversity. The resulting variance in the phenotypic distribution obscures the signal of increasing cell movement. Our habitat-destruction tumor model on the other hand does include strong selection for cell movement, which reduces phenotypic diversity and results in a strong directed increase in cell movement and invasion speed with time. Since, the environment is destroyed as cancer cells grow in a particular spatial location there is constant selection to invade the frontier, and this selection drives an accelerated invasion speed. Hence, we predict that in tumors with a narrow band of living cells and a large necrotic core invasion speed will accelerate with time.

As empirical research into tumor dynamics progresses, it will be important to determine, whether models with evolving cell motility provide better predictions of tumor growth than models without evolutionary changes. Data on tumor growth has been of low resolution and only very simple models have been fit to this data. For example, it appears that the best dynamic model we currently have to explain tumor dynamics is the power law model (Hart et al., 1998). Higher resolution data, for example data that resolves cell densities in space, and more sophisticated predictive models will ultimately progress our understanding of the mechanisms that produce patterns of tumor growth and invasion (see McDaniel et al., 2012).

In our habitat-destruction model, the wake of habitat-destruction precludes a permanent niche for a competitive phenotype. As a result, there is no spatial coexistence of phenotypes. However, the habitat–continuum tumor model generates the coexistence of the colonizer and competitor phenotypes. Furthermore, there is clear phenotypic differentiation in space. Part of this differentiation may be due to spatial sorting. Benichou et al. (2012) demonstrated the effect of spatial sorting in a very similar model. However, there is selection for movement in our model, and this creates an even stronger pattern of spatial differentiation. Furthermore, given the competition colonization tradeoff, the phenotypes are even more strictly localized in space than they otherwise would be. We predict that in tumors characterized by smaller regions of necrosis and successful angiogenesis, there will be two distinct phenotypic populations – motile and invasive cells at the tumor margin and angiogenic cells in the tumor interior.

While distinct genetic populations have been observed in tumors, there has been no attempt to determine a specific spatial distribution. We predict that spatial mapping of both clinical and experimental tumors should show invasive cellular features such as invadopodia to be most common in the tumor rim while cells expressing VEGF should be more common in tumor regions deep to the edge. Interestingly, Grillon et al. (2011) recently examined spatial distribution of a few cell membrane proteins in C6 glioblastoma tumors growing in a rat brain. They found Na^+^/H^+^ exchanger (NHE-1) and lactate-H^+^ cotransporter (MCT1) were upregulated at the tumor edge, while MCT4 and carbonic anhydrase (CAIX) were not upregulated at the tumor edge. A future research challenge in characterizing cancer cell phenotypes will be to differentiate phenotypic plasticity (changes that can occur within an individual cell) from heritable phenotypic changes (inter-generational changes).

In conclusion, we propose that understanding the role of ecology and evolutionary adaptations in tumors is necessary to fully understand tumor biology. The genetic evolution occurring within tumors is well documented, but the governing dynamics for that evolution should be strongly influenced by environmental selection forces. It is plausible that the competition colonization tradeoff that commonly influences spatial distributions of species and phenotypes in nature also influences intratumoral evolution.

## Conflict of Interest Statement

The authors declare that the research was conducted in the absence of any commercial or financial relationships that could be construed as a potential conflict of interest.
